# UAV Path Optimization for Precision Agriculture Wireless Sensor Networks

**DOI:** 10.3390/s20216098

**Published:** 2020-10-27

**Authors:** Gilson E. Just, Marcelo E. Pellenz, Luiz A. de Paula Lima, Bruno S. Chang, Richard Demo Souza, Samuel Montejo-Sánchez

**Affiliations:** 1 PPGIa-Graduate Program in Computer Science, Pontifical Catholic University of Parana, Curitiba 80215-901, Brazil; gilson.just@ppgia.pucpr.br; 2 Department of Electrical Engineering, Pontifical Catholic University of Paraná, Curitiba 80215-901, Brazil; laplima@ppgia.pucpr.br; 3 CPGEI/Electronics Department, Federal University of Technology—Paraná, Curitiba 80230-901, Brazil; bschang@utfpr.edu.br; 4 Department of Electrical and Electronics Engineering, Federal University of Santa Catarina, Florianópolis 88040-900, Brazil; richard.demo@ufsc.br; 5 Programa Institucional de Fomento a la I+D+i, Universidad Tecnológica Metropolitana, Santiago 8940577, Chile; smontejo@utem.cl

**Keywords:** unmanned aerial vehicle, path planning, precision agriculture, wireless sensor networks, data gathering

## Abstract

The use of monitoring sensors is increasingly present in the context of precision agriculture. Usually, these sensor nodes (SNs) alternate their states between periods of activation and hibernation to reduce battery usage. When employing unmanned aerial vehicles (UAVs) to collect data from SNs distributed over a large agricultural area, we must synchronize the UAV route with the activation period of each SN. In this article, we address the problem of optimizing the UAV path through all the SNs to reduce its flight time, while also maximizing the SNs’ lifetime. Using the concept of timeslots for time base management combined with the idea of flight prohibition list, we propose an efficient algorithm for discovering and reconfiguring the activation time of the SNs. Experimental results were obtained through the development of our own simulator—UAV Simulator. These results demonstrate a considerable reduction in the distance traveled by the UAV and also in its flight time. In addition, the model provides a reduction in transmission time by SNs after reconfiguration, thus ensuring a longer lifetime for the SNs in the monitoring environment, as well as improving the freshness and continuity of the gathered data, which support the decision-making process.

## 1. Introduction

Unmanned aerial vehicles (UAVs), popularly known as drones, are increasingly used in agriculture and livestock for monitoring, spraying, and tracking, as well as for imaging and sensory data collection. Advancements in UAV technology in recent years have significantly reduced costs. Higher accessibility for the technology translates into rapid dissemination of information via specialized websites and discussion forums, and the development of new features and applications for drones. Other areas, such as terrain mapping, search and rescue, security, cinematography and delivery, were revolutionized by this technology [1].

Many studies about UAV applications aim to solve society modern problems. Klaine et al. [2] proposed the deployment of an intelligent network using reinforcement learning to automatically position UAV base stations in emergency scenarios. German et al. [3], by their turn, applied the use of collaborative UAVs to create communication networks between users in disaster situations. Mayor et al. [4] formulated a new problem for the optimal placement of UAVs geared towards wireless coverage provision for voice over WiFi service to a set of ground users confined in an open area. Andrade et al. [5] use a collaborative network created by UAVs for search and rescue missions, achieving success rates 50% higher than those led by just a single UAV. Lee et al. [6] created an optical system using a laser to guide UAVs in urban environments so that they can land safely in case of failures on GPS signal reception. Cerba et al. [7] studied the application of a radiation monitoring system through sensors in nuclear power plants, citing Fukushima in Japan as an example. They adopted the use of UAVs to collect this sensory data at monitoring points. Zhang et al. [8] created a route planning system for UAVs to patrol highways and urban environments, achieving up to 16% of path reduction when compared to conventional vehicles doing the same job. Hireche et al. [9] presented a scalable approach to model uncertainties within a UAV embedded mission manager; this proposal considers the context of the mission including external constraints, the health of the UAV, and the computing resource availability. Yang et al. [10] used a collaborative network of UAVs to assist in the processing of IoT devices activities that are computationally limited. Jayme et al. [11] applied the use of UAVs to monitor livestock through aerial images, using machine learning techniques to distinguish between different breeds of cattle.

Estimates made by the World Bank indicate that in 2050 there will be about 9.7 billion human beings living on our planet, demanding an increase of 70% in agricultural production [12]. There is no doubt that the use of technology will bring greater productivity to this sector. In [13], Maksimovic et al. address how technology can help in food production, protection, processing, packaging, transportation, and traceability. As an example, a producer carrying a backpack sprayer takes a whole day to spray one hectare of land, while a drone equipped with a spraying system can perform the same job in just one hour [14]. Furthermore, there are more than three million cases of pesticide poisoning and disease in farmers registered by the World Health Organization (WHO) in India alone [15], a situation that can be easily resolved with the use of UAVs. Drones have better precision during poison application, avoiding waste and direct handling of toxic substances by human beings. Drones are also able to take high-precision pictures in certain points of the plantation, analyzing points of failure in the harvest and giving more information to farmers. In addition, the data collection time is key in the decision-making process, since IoT agricultural networks sometime provides varying measurements; therefore. it is necessary to ensure that every message is fresh [16].

The correct measurement of the water level present in the soil is fundamental for agriculture [17]. The management of water resources is essential to control soil humidity. The excess of water generates a loss of nutrients, causes root rot, as well as water stress for plants. Many of these processes are still manual, requiring that a worker goes to certain points of the plantation to gather the information required to decide whether or not to irrigate the soil at those points. Developing countries, which have agriculture as their main source of wealth, still have many of their sensory data gathering processes carried out manually [18].

The use of UAVs to collect sensory data has been widely researched due to the need to increase productivity in the sector. Kashuba et al. [19] carried out studies to optimize UAV paths using the bisector angle to direct the UAV through sensor nodes (SNs), reaching UAVs flight path optimization rates of approximately 25% compared to a non-optimized route, where the UAV must fly over all SN coordinates. Trotta et al. [20] studied the use of UAVs to collect sensory data scheduling the active time of grounded SNs with UAV flight enhancing SN lifetime. Xiong et al. [21] studied a model that synchronizes the UAV path according to the transmission and hibernation period of sensors on the ground, seeking energy efficiency improvements for stations. Zhang et al. [22] researched the application of UAVs for the collection of sensory data, also synchronizing the hibernation and transmission period with SNs. However, they focused their studies on the communication channel model with the UAV and an in-depth detailed analysis on transmission rates and signal strength at the interfaces of the elements involved. Olivieri et al. [23] investigated the advantages of working with collaborative UAVs gathering sensory data using DTNs-Delayed and Disconnection Tolerant Networks, besides the advantages that this communication model adds to the system. The authors also reported several benefits in using UAVs when compared to land vehicles in agriculture, and proposed route optimization models for problems such as the traveling salesman problem (TSP) and the vehicle routing problem (VRP).

Although there are many studies on the application of UAVs in agriculture for collection of sensory data, many authors point out the difficulty in designing systems where SNs have their transmission and hibernation intervals scheduled according to the predefined UAV route [20,24], still being a challenging issue.

The main objective of this work is to propose a new strategy where we displace SNs over a large area with no direct communication between them. Then, we consider a UAV as a mobile sink to gather data, where the SN has it own timeslots to become active. The SN’s active timeslot can be shared or dedicated. As the UAV must fly over them to find out which are their active timeslots, along with the Prohibition List, we propose a new strategy ensuring UAV path length reduction, associated with a reduction in the SN active time after SNs are properly scheduled. Our focus was not to evaluate the quality of data transferred between UAV and SNs, or study energy constraints of the communication because these aspects were already explored by related authors [20,21,22]. Our goal in this work is to investigate the dynamics of UAV flight to understand which scenario presents better results based on each functionality of the system.

The rest of this paper is structured as follows. Section 2 presents the characteristics of the UAV path optimization models and main system elements. In Section 3, we present the proposed strategy for the UAV algorithm during the SN’s reconfiguration phase, and the functionalities of the so-called Prohibition List for a more accurate flight and management. In Section 4, we explain the reasons for the development of a new simulation platform, introduces the main functionalities of the UAV Simulator, and we discuss the methodology used to obtain the results disclosed by our research. In Section 5, we present the average results obtained for each simulation scenario and a detailed analysis of the proposed model. Finally, in Section 6, we discuss the conclusions and future works.

## 2. System Model

For this work, we consider that the sensory data generated by SNs are gathered by a single UAV used as a sink node [25], as it flies over all wireless SNs. Our objective is to deliver all the collected data to the Base Station (BS), which is the final destination and point of concentration of all sensory data for future analysis. First, we introduce the main elements of this system, detailing the main functionalities of the UAV, Sensor Nodes, and Base Station. After that, we discuss an optimized path for the UAV to fly over the SNs, minimizing the total flight.

### 2.1. System Elements

**Figure 1:**
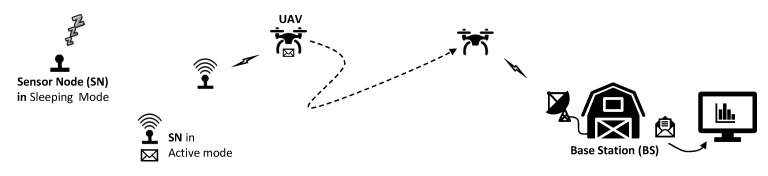
System elements.

As illustrated in Figure 1, the scenario considered in this work is composed of SNs, UAVs, and a BS, while we consider the following characteristics for each element:

Sensor nodes (SNs) are homogeneous, have fixed locations, and are known by the UAVs. We assume that they are composed of a set of sensors, processor, memory for sensory data storage, and also a Global Positioning System (GPS) interface. Besides that, we consider that SNs have a limited lifetime due to the capacity restrictions of their batteries. Thus, SNs must alternate their states between periods of activation and hibernation. We assume that there is no direct communication between SNs due to the distance between them and the limited communication capacity. Therefore, they depend on the UAV to collect data stored in memory and deliver it to the BS.

Unmanned aerial vehicles (UAVs) have high mobility, are homogeneous, multi-rotor, and have ad hoc communication systems [26] for UAV-UAV, UAV-SN, and UAV-BS communication type. Besides gathering data from the SNs, they can exchange information with each other by copying its local database, about the scenario, directly to the new UAV on Return to Home procedure, and transfer the sensory data to the BS.

Base station (BS) has a fixed location known by the UAVs, and it is the final destination of the information generated by the SNs. It is primarily responsible for the post-processing of the sensory data delivered by the UAVs.

### 2.2. Path Planning

The first step for gathering sensory data from SNs is to perform the UAV path planning. Thus, it is necessary to previously define a route between them, so the UAV must fly over each SN coverage area. However, mathematically there are countless potential trajectories, making it possible to create more economical routes in terms of flight distance. The TSP algorithm that provides the lowest path cost among all SNs has NP-Hard [20,27] complexity; therefore, several studies propose heuristic models that may offer results as good as TSP, but with a much lower computational complexity. We used as a benchmark the Nearest Neighbor (NN) heuristic TSP model without optimization, which forces the UAV to fly over the coordinates of each SN. In our research, we specifically adopted the Directional NN Algorithm Directed to the Next Nearest Node—DDNN route optimization model. According to the author in [28], the NN offers the most direct representation of a heuristic model for the TSP which generates fairly close results to the optimal route. The DDNN model, on the other hand, still offers greater reduction due to the fact that it uses the limit of the SN communication radius as an entry point to the UAV.

The DDNN [28] path optimization model calculates the current UAV position in a straight line to its next sensor, always using the intersection of its trajectory with the SN coverage radius. The model always adopts the closest sensor to the UAV current position as next hop. The algorithm implementation is simple and has a low computational cost for the UAV, which is important since it is necessary to constantly reschedule the route as it flies over the SNs. The result of the application of the DDNN model generates a pair of coordinates called *nearest point* ($N_{p}$).

**Figure 2:**
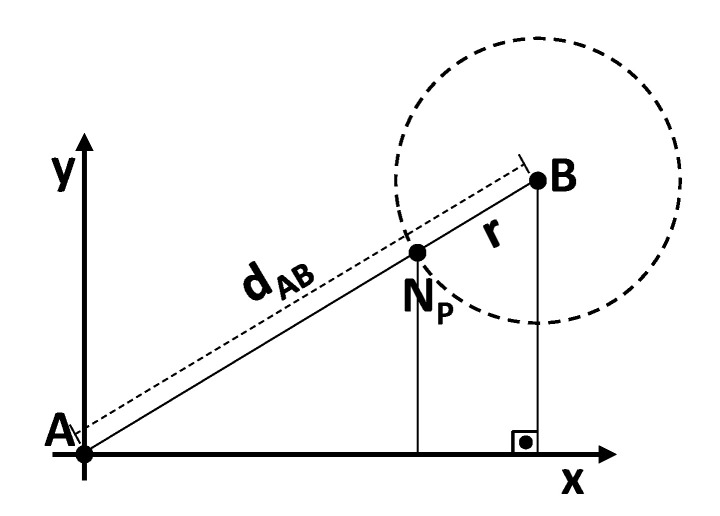
*Nearest point* geometrical representation.

Figure 2 illustrates the *nearest point* geometrical concept, where we assume that the UAV is located at the *A* vertex of a right triangle, whose coordinates are defined by $A=(a_{x},a_{y})$. We can calculate the Euclidean distance for the next SN located at point $B=(b_{x},b_{y})$, $d_{A,B}=\sqrt{{\left(b_{x}-a_{x}\right)}^{2}+{\left(b_{y}-a_{y}\right)}^{2}}$. Note that this is an approximation (although very tight) for the flight distance calculation because we are considering very low altitude flight. The nearest point $N_{p}$ is located between vertices *A* and *B*. The $n_{x}$ and $n_{y}$ coordinates of the *nearest point* are calculated using the Thales intercept theorem [29],

(1) $$n_{x}=a_{x}+\frac{\left(b_{x}-a_{x}\right)\left(d_{A,B}-r\right)}{d_{A,B}}\;\;\;\;\;\;\;\;\;\;\;\;\;\;\;n_{y}=a_{y}+\frac{\left(b_{y}-a_{y}\right)\left(d_{A,B}-r\right)}{d_{A,B}},$$

where the parameter *r* denotes the SN communication radius. However, if we consider higher altitudes, it is important to take the UAV flight height on the equation.

**Figure 3:**
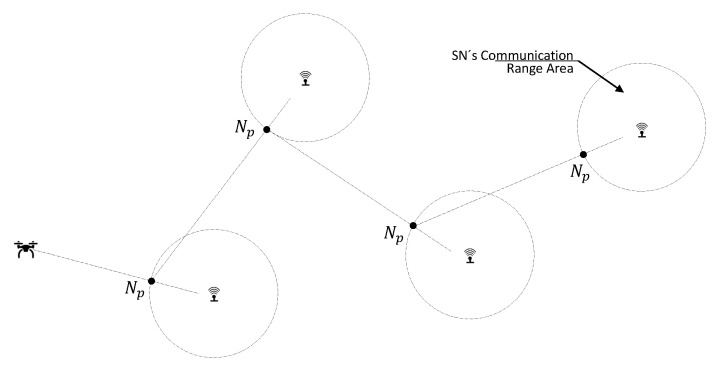
Example of path created by the DDNN model for UAV.

Using the DDNN model, we can calculate the UAV flight distance ($D_{n}$), from its origin to the *n*-th sensor ($S_{n}$). The flight distance is

(2) $$D_{n}=\sum_{i=1}^{n}d_{i-1,i}$$

where $d_{i-1,i}$ is the distance between the (i−1)th and *i*th SNs, except $d_{0,1}$ which is the distance between the UAV initial position (BS location) and the nearest point of its first target destination (SN closest to the BS). Figure 3 illustrates the nearest points ($N_{p}$) and the resulting pre-calculated UAV route following the DDNN model. The authors in [28] performed comparisons between different heuristic TSP algorithms. Remarkably, they claim that the DDNN model presents a good trade-off between complexity and path delay reduction.

## 3. Proposed Strategy

The proposed strategy for reconfiguring the SNs aims to synchronize their activation and hibernation periods with the UAV flight. It permits SNs to transmit for the shortest time, thus reducing the energy consumption from their batteries, and maximizing their lifetime [8,21,24,30]. UAVs, like SNs, use timeslots as a time reference for the management of their states.

### 3.1. Timeslots as Time Base

We adopt as a premise large territorial extensions where there is no signal coverage by a cellular network or even other internet service providers. Therefore, SNs may use GPS signals to adjust and synchronize their internal clocks, a viable alternative that is increasingly cheaper and has low energy consumption [31,32].

As an alternative method, we consider an initial configuration of the slotframe and timeslot time for each SN before its deployment. However, in this approach, there is a problem of synchronism drift among the clocks of all SNs, which must be corrected during the UAV flight phase. This can be done by the UAV sending two rendezvous messages, one at the beginning and another in the middle of the timeslot period. This procedure ensures synchronization with jitter of up to half the duration of a timeslot [33]. If the UAV specifies, in these association messages, when it is the beginning and when it is the middle, the sensors will be able to align their timers in relation to the start of a timeslot. If the UAV also sends the number of that timeslot within the slotframe; then, both corrections are successful.

Furthermore, we assume that, when starting the UAV flight, each SN finds itself in a random timeslot, unknown by the UAV. In other timeslots in which the SN is not in active mode, we consider that they are in hibernation mode. The total flight time to collect the data from the *N* SNs assigned to a specific UAV is

(3) $$T_{N}=\sum_{i=1}^{N}\left(\frac{d_{i-1,i}}{v_{a}}+t_{i}\right)=\frac{D_{N}}{v_{a}}+\sum_{i=1}^{N}t_{i}=\frac{D_{N}}{v_{a}}+Nt_{R},$$

where $D_{N}$ is the UAV total flight distance to cover the *N* SNs distributed throughout the total area assigned to the UAV, $v_{a}$ is the average UAV speed, and $t_{i}$ is the communication time between the *i*th SN and the UAV. We assume the same communication time between UAV and *i*th SN, denoted by $t_{R}$.

**Figure 4:**
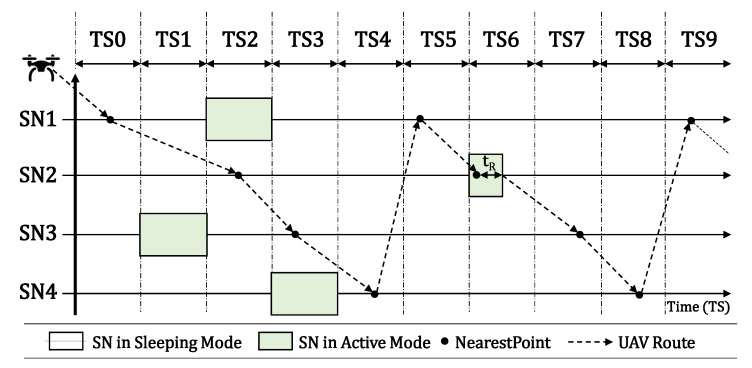
Individual Timeslots scenario.

**Figure 5:**
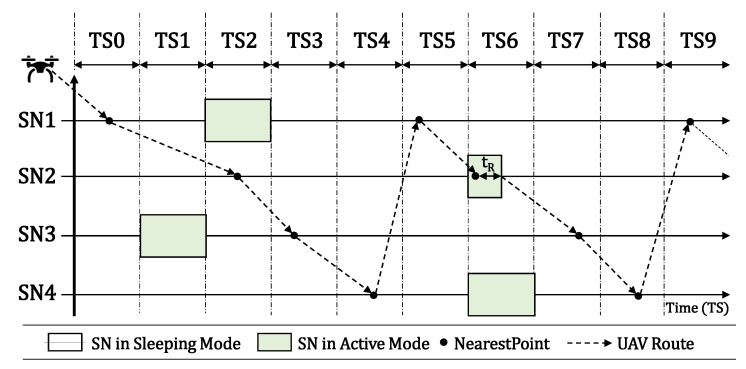
Shared Timeslots scenario.

In individual scenarios, we assume that each SN has its own timeslot to become active, as presented in Figure 4. In shared timeslots’ scenarios, we assume that two or more SNs can become active at the same timeslot, as presented in Figure 5. In both scenarios, the process is the same, described by flowchart 1, boxes 7, 8, and 11 to 14, in Figure 11a. The UAV flies over the SNs to locate and reconfigure them. When the UAV finally finds SN2 active over TS6, it reads its data during a period called Reading Time ($t_{R}$) here, reconfigure its activation timeslot according to its own route, and then shuts it down to hibernate again until its new timeslot.

The main difference between the two scenarios is that with individual timeslots we assume one SN per timeslot and the UAV blocks that TS for a future fly in it Prohibition List. In scenarios with shared timeslots, the UAV keeps flying over other SNs. We can observe in both figures that the SN activation time becomes shorter when it was already found by the UAV, since it stays in sleep mode until the next UAV visit.

**Figure 6:**
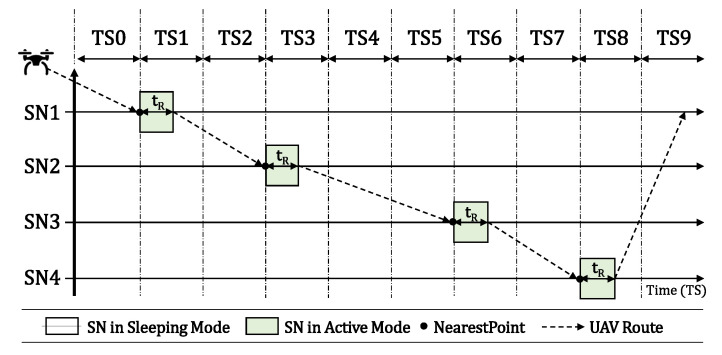
SN activation timeslot scheduled according to the UAV flight.

Figure 6 illustrates the final result after all SNs have been properly reconfigured by the UAV. Each one is now transmitting on its scheduled timeslot, according to the UAV flight. The UAV that is in its initial position at the BS must follow its pre-defined route among all SNs, so that each SN has its own timeslot for starting their active state. As we can see in Figure 6, the UAV must travel to SN1 and the time for this displacement is one TS. Therefore, SN1 must start its active mode in TS1. After successfully connecting and reading the sensory data from it, the UAV shuts it down to hibernation state for a calculated time (in timeslots), so the UAV can fly to its next destination. After that, the UAV has more than one timeslot to fly from SN1 to SN2, which will be in active mode only in TS3. Thus, after the UAV collects the sensory data from SN2 and shuts it down, the UAV flies to SN3, which is distant from its current position. Consequently, it needs more than two TSs to displace to its new target, so SN3 must be configured to be active only on TS6. This procedure is repeated between SN3 and SN4, and the UAV finds SN4 transmitting on TS8.

Changes in the topology and SNs redistribution often occur. The impact caused by SN addition, removal, or moving directly affects (3), as they change the total UAV flight distance and its flight time. The insertion of SN in new points will often be necessary, due to the decision from the agronomists responsible to detect blind spots in the analysis of the crop, or even due to issues of territory expansion. Furthermore, the removal of sensors for maintenance purposes or even the exchange of sensors may also be considered. Therefore, for such situations, we treat it as a new scenario.

**Figure 7:**
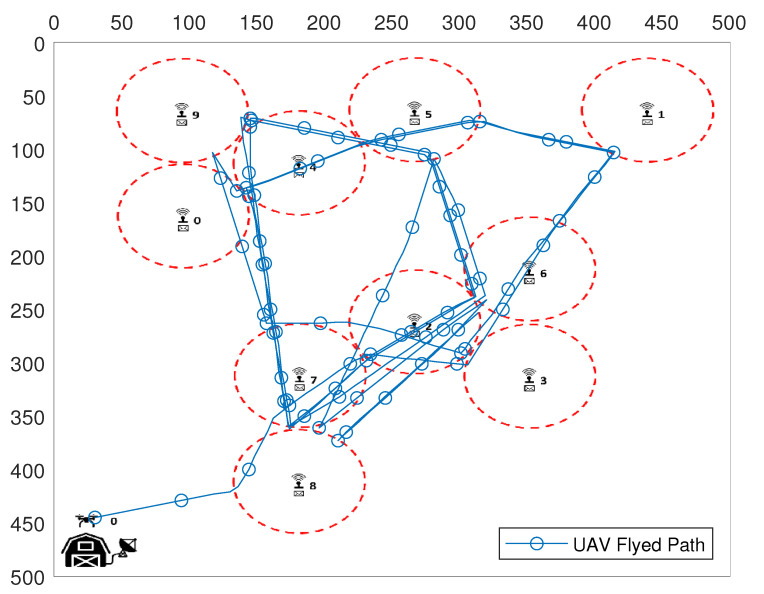
UAV trajectory to reconfigure all SNs.

**Figure 8:**
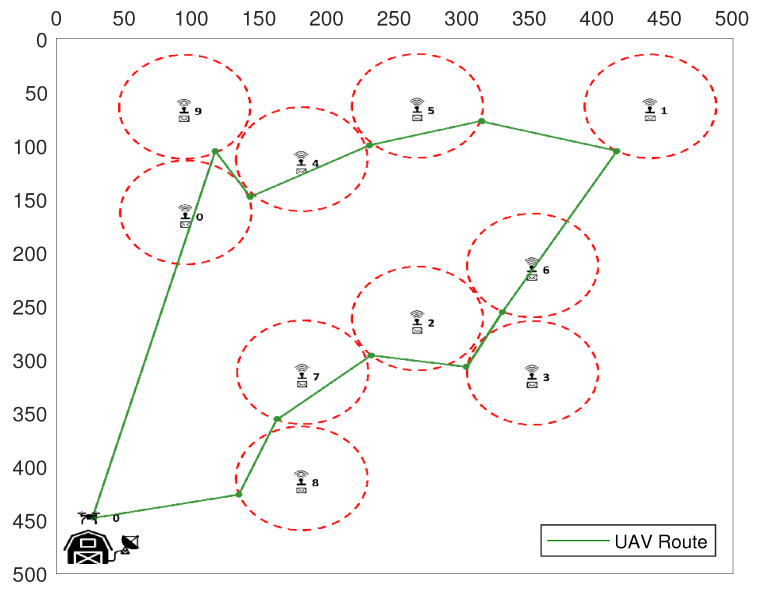
UAV route after SNs are configured.

Figure 7 and Figure 8 show a simple scenario created by the UAV Simulator with 10 SNs, in an area of 500 × 500 m, and with SNs communication range set to 50 m. In Figure 7, we have the total flight path representation taken by the UAV during the SNs reconfiguration phase. As shown in Figure 8, after all SNs were reconfigured, each SN active time is scheduled according to the UAV flight path.

### 3.2. Prohibition List

In order to efficiently direct the UAV between SNs, we propose a Prohibition List algorithm, which is managed by the UAV itself and aims to minimize the displacements towards SNs that are in hibernation mode. During the initial phase of SN reconfiguration, the UAV increases this list as it flies over the active or hibernating SNs. The objective is to enable the UAV to consult its list in each displacement, avoiding going through the SN in already known TSs (where they are in hibernation state) and directing the UAV to other SNs, by changing its own route.

**Figure 9:**
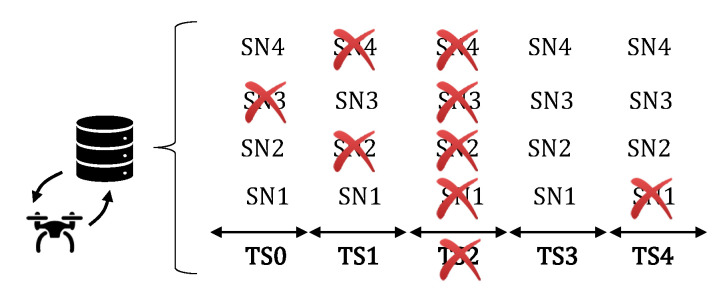
Example of the Prohibition List managed by the UAV during the reconfiguration phase.

Figure 9 illustrates the Prohibition List created and used by the UAV after a certain SN discovery phase. Note that in TS0 the UAV has already passed through SN3, so the UAV disregards this possible destination in the next discovery round, making a decision to move to another pending SN on its route, or to wait on standby until the next timeslot. We can also observe that TS2 is no longer considered valid by the UAV. This is due to the UAV having already located an active SN on that timeslot. Therefore, for scenarios where timeslots are dedicated the UAV automatically disregards all SNs during this period of time; for scenarios where timeslots are shared between the SNs, it keeps flying because there is the possibility to find another SN active for that timeslot.

### 3.3. UAV Flight Strategy

**Figure 10:**
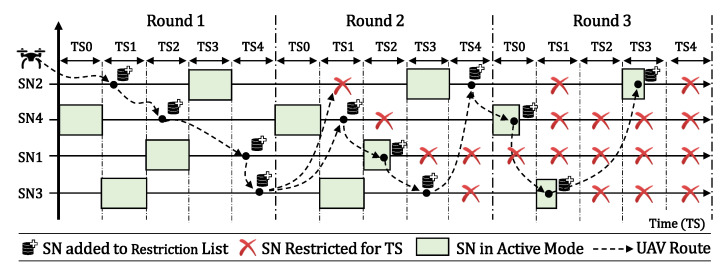
Graphical representation of the UAV search strategy.

Figure 10 shows the UAV flying over the SNs following its optimized DDNN route, BS → SN2 → SN4 → SN1 → SN3. After that it returns to SN2, starting again the discovery cycle until all SNs are properly reconfigured to their new timeslot. The UAV reaches its first target SN2 during TS1, and SN2 was not found in active mode in this timeslot. Therefore, SN2 is added to the Prohibition List for TS1. Thus, in the next round during TS1, the UAV will fly over this SN. The UAV also passes through SN4 during TS2, and adds it to its Prohibition List for that timeslot. The UAV follows its route to SN1 where it arrives at TS4, and also adds to its prohibition list. Finally, it arrives at SN3 still in TS4, does not find it in active mode, and also adds it to the list. Once the route is over, the UAV restarts its route and calculates the arrival TS for SN2. By checking its database list, it discovers that the SN is prohibited for TS1. Therefore, the UAV takes a decision and heads to its next destination, thus avoiding passing over an inactive SN at that TS. As the UAV acquires knowledge about the scenario, it starts managing its own route based on these list, making more efficient decisions in relation to which SN it should proceed next.

Moreover, the UAV has the ability to calculate the arrival TS until the next SN, validating this information with its Prohibition List. Thus, it checks whether or not the next SN is classified as restricted for the calculated TS. If the destination is prohibited, the UAV continuously performs this calculation until there is an SN available for the TS. If there is no SN available, the UAV waits on its current position until next TS, performing the calculation for each pending SN in its list.

**Figure 11:**
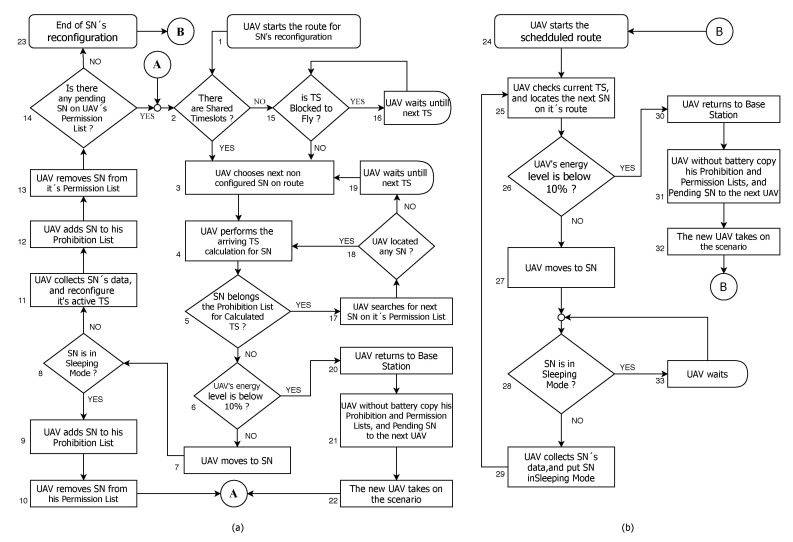
(**a**) strategy adopted by UAV for SNs reconfiguration phase; (**b**) strategy adopted by UAV after the SN reconfiguration phase.

The flowchart shown in Figure 11a represents the UAV strategy during the SN reconfiguration phase. Figure 11b represents the UAV strategy after the completion of the SN reconfiguration stage, where the UAV follows its route visiting only SNs in active state.

In Figure 11a, steps 2, 15, and 16 represent the UAV decision points. For the dedicated TS method, there is no way that two or more SNs are active during the same TS. Thus, since the UAV locates an SN over TS2, for example, there is no longer a need to fly over searching for other SNs during that timeslot on future rounds. Therefore, the UAV waits on its current position until the next free timeslot (step 16). For scenarios with shared TSs between SNs, there is the possibility of having two or more devices active during the same TS. Thus, the UAV continues flying its route during that timeslot in which the UAV has already found one or more active SNs. Then, the UAV targets the next SN from its route (step 3) and calculates the arrival TS (step 4). The UAV checks its Prohibition List for the calculated TS, if the SN is restricted or allowed for that TS (step 5). If there is no restriction, the UAV checks its energy autonomy (step 6) and, if there is enough battery charge, it moves to its new target (step 7).

Upon arriving at the SN NearestPoint/DDNN coordinates, the UAV checks if it is in hibernation mode or not (step 8). If the SN is inactive, the UAV adds the SN to its Prohibition List for current TS (steps 9 and 10), and resumes its route to the next SN (step 2). If the UAV finds the SN in active mode (step 8), then it collects the sensory data from the SN, and reconfigure it to the calculated TS according to its route (step 11). The UAV then updates its Prohibition List (steps 12 and 13) and checks if there are any pending SNs (step 14). If so, the UAV resumes its initial SN search state (step 2). Otherwise, the UAV finishes the SN reconfiguration phase (step 23) and proceeds to scheduled flight according to each SN state (step 24).

During the entire UAV journey between the SNs, the autonomy of its battery is continuously checked (step 6). If it gets below a critical level (as for instance 10% [34]), the UAV returns to the BS (step 20) and transfers its Prohibition List and pending SN list to a new UAV (step 21) that will take the route (step 22), returning to step 2 in the flowchart.

After the reconfiguration phase (step 23), we present the flowchart in Figure 11b, where the UAV starts flying according to the SNs scheduled states for each timeslot (step 24). The UAV initially checks the current TS, searches for the next available SN (step 25), and consults its battery charge (step 26). If there is enough autonomy, the UAV then heads for the SN (step 27). If the SN is already in active mode (step 28), the UAV collects its sensory data, and shuts it down until next round (step 29), flying to the next SN (step 25). When the UAV arrives, if the SN is still hibernating (step 28), then the UAV waits on standby mode (step 33) until the SN becomes active again. Even so, after the SN reconfiguration phase, the UAV autonomy control is still checked (step 26), always taking into account that it can be replaced by another UAV, which will assume the data collection through steps 30–32 from the flowchart.

For the sake of simplicity, and without loss of generality, we assume that (i) when each SN wakes up its data are ready to be gathered by the UAV (few bytes); (ii) the transmission time of this information is negligible compared to the defined timeslot duration; and (iii) the cumulative impact of these times can also be neglected when considering the average battery life, since the total collection would not exceed the unit of kilobytes of information.

## 4. Simulation Methodology

In this line of study, due to the high cost of autonomous UAV platforms, it is very common to use simulators for mathematical validation of the route models, and to analyze the experimental results generated through them. Many researchers use general purpose systems such as Network Simulator [35], Matlab [36], The One [37], or even OMNet++ [38] for results generation and analysis. Furthermore, there are open source platforms developed specifically for UAV simulations, such as the OpenUAV [39] project, or even the Dronecode [40] that provide a library for graphical development applied to this area. Still, several authors choose to develop their own simulators instead of using a ready-made solution.

**Figure 12:**
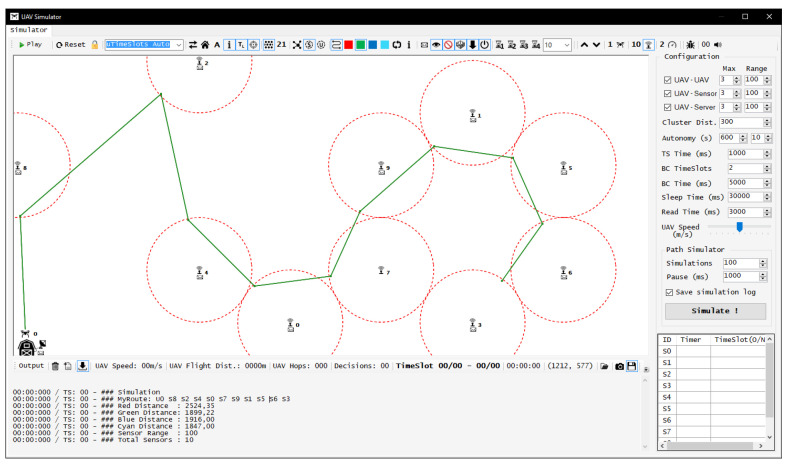
UAV Simulator main interface presenting the UAV’s path between the SNs.

We have followed the latter path and chose to develop our own graphic simulator: UAV Simulator. Our platform was developed using Visual Studio 2017 as IDE and C-Sharp language; the source code is freely available at GitHub [41]. The reason for the development of a new system was the creation of a graphical interface that allows for the real-time monitoring of the UAV flight path, along with the activation and hibernation status from each SN in the scenario. Besides that, it provides the visualization of the ad hoc connections created by the approximation between UAV and SN/BS, and also permits easy monitoring of the sensory data transfers from SN to UAV, UAV to UAV, and UAV to BS in a clean and intuitive interface to the viewer. The simulator also allows the generation of logs of all states and elements involved in the scenario, ensuring detailed and accurate information for further analysis, and study of the proposed model. Figure 12 shows the main interface of the UAV simulator [41].

In terms of features and functionality, the graphical UAV Simulator allows the use of up to 30 SNs, along with up to 30 UAVs; resizing of the simulation area is also possible. It allows configuration of the communication range and connections from the UAV with SNs, other UAVs and BS. The management of the UAV autonomy by flight time, the SNs active time, and UAV time over each SN to collect the sensory data (called here as Reading Time) are also configurable. The simulator has real-time monitoring of the active and hibernation timers from each SN, and their assigned timeslots. Furthermore, the simulator has a text panel, informing the viewer of each step and decision made by the UAV, and individual events from all SNs. For each simulation scenario, the simulator registers the following information:

- Number of UAVs needed to reconfigure all SNs;
- Flight distance of each UAV;
- Flight time of each UAV;
- Total flight time;
- Number of hops by each UAV;
- Number of decisions taken by each UAV;
- UAVs pre-calculated path length;
- Timeslots assigned to the scenario;
- Timeslots elapsed until all SNs are reconfigured;
- UAV coordinates as a function of flight time/TS;
- SNs states as a function of time/TS;
- UAVs connections with each SN as a function of time/TS;
- SNs missed flights by UAV;
- SNs Active Time (before and after reconfiguration);
- Battery percentage and UAV autonomy as a function of time/TS.

The proposed strategy was evaluated through simulation, considering five different scenarios ($S_{1}$, *…*, $S_{5}$), which are presented in Table 1. The parameters adopted for flight autonomy and the UAV speed were based on the DJI manufacturer (DJI, Shenzhen, Guangdong, China) [42], Phanton 4 Pro, Matrice 200 and Mavic 2 Enterprise models, recommended for use in agriculture due to the ease of its graphical interface mapping and flight plan. The flight time of the mentioned UAVs varies between 10 and 30 min, depending on the flight speed, used resources, and weather conditions. Furthermore, authors such as Yang et al. [27] and Maxa et al. [43] adopt 10 min of flight autonomy in published studies. Authors in [8,44,45] also cite UAVs in the context of sensory data gathering operating at speeds between 5 and 40 m/s. As multi rotor UAVs has high-mobility model, we assumed a fast deceleration process during the reading time interval. As the UAV is already inside the SN’s communication range, it begins the communication process during this deceleration.

In the first scenario, $S_{1}$, we set the UAV average speed to 25 m/s. We also assume that there is no possibility that two or more SNs are active on the same TS, so we activate the *dedicated timeslots system*. Even so, we activate the UAV learning strategy, ensuring that its displacements between one SN to another are in accordance wth the Prohibition List. Thus, when moving to the next SN, the UAV first checks if the current TS is free. If positive, the UAV flies to the next SN; otherwise, it moves to another SN that is not in the Prohibition List. If there are no possible targets, the UAV waits on its current position until the next timeslot.

In the second scenario, $S_{2}$, we disregard the use of dedicated TSs, that is, they may be shared among SNs. At the moment, we start the simulation, each SN receives a random TS assigned to it and there is a probability that at that moment, one or more SNs start transmitting during the same TS. In addition, we have adopted the strategy of Prohibition List that guide the UAV destinations, and also we set up its average speed to 25 m/s. The purpose of the comparison between scenarios $S_{1}$ and $S_{2}$ is to measure the impact caused on the UAV regarding the sharing of TSs between SNs.

For the third scenario, $S_{3}$, we assume a passive SN discovery procedure, where the UAV waits hovering over each SN until it changes to the active state, so it is reconfigured for the TS according to its route for those coordinates. Then, the UAV flies to the next SN in its route and waits again over it, until the SN changes its state to active mode, and so on until all pending SNs configuration is completed. When compared to the first scenario, $S_{1}$, this scenario aims to identify whether the active mode of SN search is more effective than simply waiting over each SN. For scenario S3, the displacement of the UAV is similar to its original route; therefore, despite the UAV moving less between SNs its average speed is set up to 25 m/s between one SN and another. Furthermore, it is not possible for the UAV to increment and use its Prohibition List due to its waiting time over a single SN while timeslots are passing by; so, it learns information about the states of a single SN.

In the fourth scenario, $S_{4}$, we disable the UAVs learning strategy, maintaining the average UAV speed at 25 m/s. Therefore, we can measure the impact of the Prohibited List and of the decision-making process with respect to the first scenario. A fundamental factor for the whole process is the flight time of the UAV, which directly impacts on its autonomy.

Finally, in the fifth scenario, $S_{5}$, we reduce the SN activation time to a single TS, in order to assess the impact of their reduced availability during the reconfiguration phase. Furthermore, we use dedicated TSs and the UAV average speed is set to 25 m/s. The reduction from the SNs active time has a direct impact on their energy consumption and autonomy; moreover, it makes its search by the UAV harder. Therefore, our goal is to measure the impact over the UAV and SNs with respect to the first scenario.

In addition to these five scenarios presented above, we generated results for other scenarios with the UAV average speed set to 15 m/s, with shared and dedicated TS models. However, as the results were very close to those for the above scenarios, we have chosen not to include them next for the sake of brevity.

In order to facilitate the comparison between our proposal and the works in [20,21,22], we present Table 2 to show the main characteristics of each work. We also explain why it is not feasible to make direct comparisons of our results with theirs in the next Section.

Our project considers scenarios where SNs are placed over a large area with no direct communication between them, so we considered a single UAV as a mobile sink to gather data. In these scenarios, the SN has it own timeslots to become active (as considered by other authors), but we propose that the SN timeslots can be shared or dedicated. As the UAV must fly over them to find out which are their active timeslots, along with the Prohibition List, we propose a new strategy ensuring the UAV path length reduction, associated with a reduction in the SN active time after SNs are properly scheduled. In addition, the UAV decisions strategy is based on always consulting the Prohibition List database. Our focus was not to evaluate the quality of data transferred between UAV and SNs, or study energy constraints of the communication because these aspects were already explored by other authors, as cited before. Our focus in this work is on the dynamics of UAV flight to understand which scenario presents better results based on each functionality of the system.

## 5. Results

For each scenario ($S_{1}$, *…*, $S_{5}$), we carry out $2\times 10^{3}$ simulations for scenario, running a total $10^{4}$ simulations for the presented average results. The descriptive results are presented in Table 3. The table presents the main evaluated metrics for the five scenarios. For each scenario, we considered 5, 10, 15, and 20 SNs. In the first column, UAV’s Battery Consumption represents the normalized UAV’s consumed battery to finish the reconfiguration scenario. The value 0.39 (Scenario $S_{1}$-5 SNs) represents UAV’s battery consumption to finish it, so only one UAV was necessary to do it. The value 1.80 (Scenario $S_{1}$-20 SNs) represents that 100% of the battery of the UAV was consumed, then it returned to the base station, and a new UAV was used to complete the reconfiguration, consuming 80% of its battery to finish it, totaling two UAVs. The Decisions column represents the number of decisions taken by the UAVs considering the Prohibition List, in order to change its own route. The Flight Distance column represents how many kilometers were taken by UAVs flight to reconfigure all SNs, and, finally, the Flight Time column represents the total time (in minutes) taken to finish reconfiguration of all SNs.

**Figure 13:**
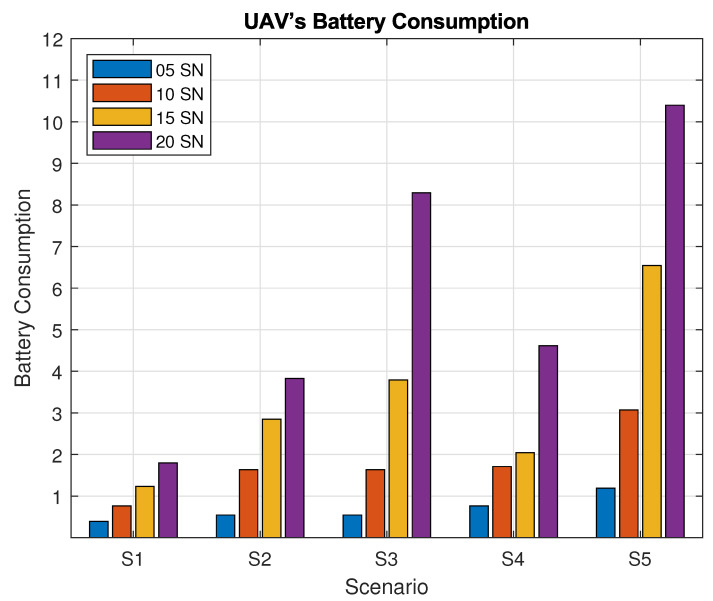
Number of UAVs used in Simulation.

Figure 13 shows how many UAVs were necessary for each scenario during the reconfiguration phase. As we can see, there was a similar usage of UAVs for cases with 5 and 10 SNs, where the SN density is lower. Regarding scenarios $S_{1}$ and $S_{2}$, it is possible to notice the difference in the amount of UAVs for 15 and 20 SNs, due to the fact that they do not share timeslots, forcing the UAV to fly over greater distances. In scenario $S_{3}$, where the UAV waits on each SN until it changes to active mode, we had a larger number of UAVs used for environments with 15 and 20 SNs. In scenario $S_{4}$, where the UAV learning system is disabled, we can observe slightly higher values compared to $S_{1}$ for 5 and 10 SNs. Finally, in the fifth scenario ($S_{5}$), we have a higher UAV occupancy compared to $S_{1}$, $S_{2}$, and $S_{4}$, due to the reduced availability of the SN to a single timeslot.

**Figure 14:**
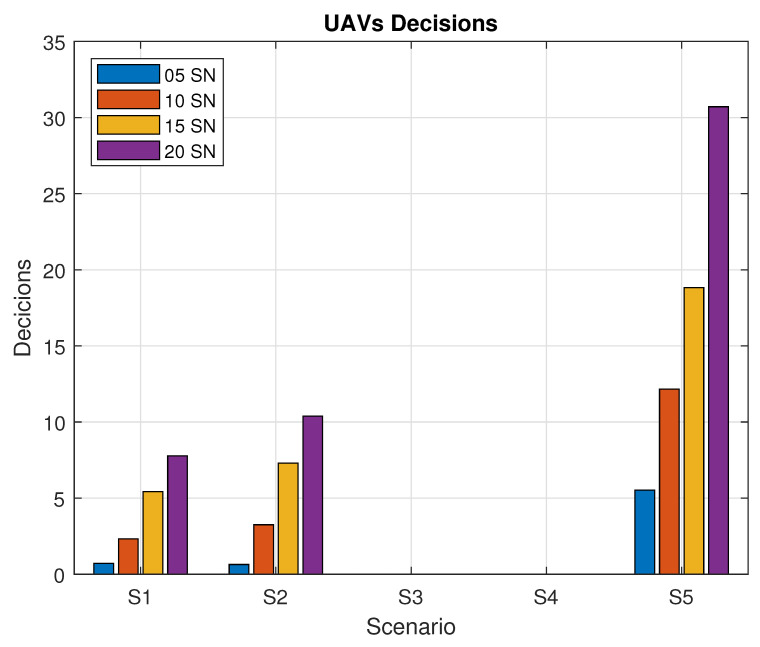
Number of decisions taken by the UAVs.

Figure 14 shows the average number of decisions taken by the UAVs for each scenario during the reconfiguration phase. A UAV decision can be to wait until the next timeslot and maintain its own position, or to go to another SN from its route list, due to restrictions for the calculated timeslot. In scenario $S_{2}$, where the TSs are shared between SNs, the UAV makes more moves; thus, it acquires the desired knowledge faster when compared to the $S_{1}$. In the third scenario, $S_{3}$, the fact that the UAV needs to wait on a single SN until it changes to active mode makes it that the UAV does not acquire knowledge regarding other SNs at different timeslots. Therefore, the UAV is not able to use its knowledge base to make decisions to improve its route. Regarding $S_{4}$, we opted to disable the Prohibition List strategy to measure the flight time and distance when compared to $S_{1}$, so no decisions were made by the UAV. Finally, in $S_{5}$, the reduction of the SN active time lowered the probability of the SN being found by the UAV; therefore, the UAV keeps continuously searching for SNs, flying over more targets, acquiring more knowledge, and making more decisions to change its own route.

**Figure 15:**
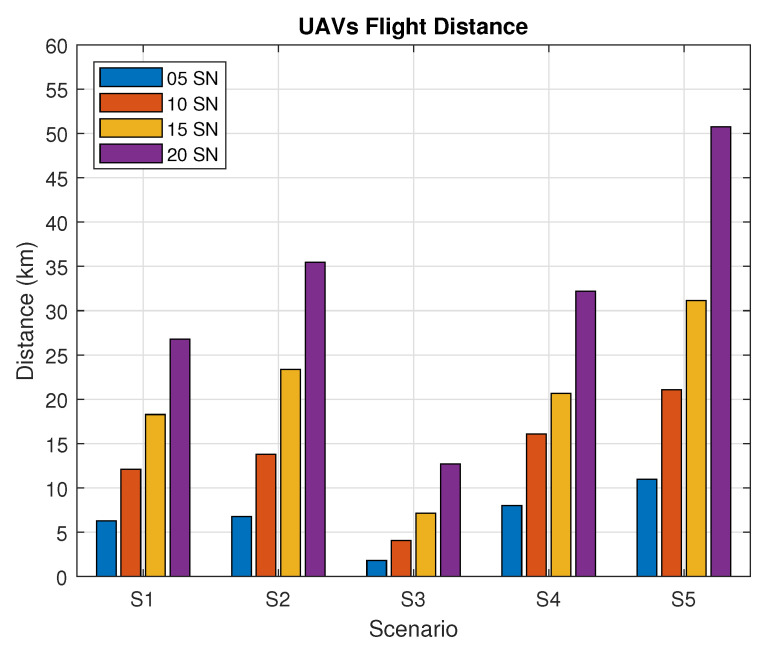
Flight path for each scenario.

Figure 15 shows the average UAV flight distance for each scenario, during the SN reconfiguration phase. In $S_{1}$ and $S_{2}$, we can see close average values for 5 and 10 SNs. In $S_{3}$, the UAV flies over each SN and hovers over it one by one. Thus, the flight pattern when compared to other scenarios is different. Thus, in this scenario, the average distance traveled by UAVs is close to the pre-calculated route at the beginning of the reconfiguration phase, with exceptions for substitutions due to the UAV autonomy. In $S_{4}$, with the Prohibition List disabled, it traveled longer distances in comparison to $S_{1}$, as expected. The most significant difference was found in environments with 15 and 20 SNs. Finally, for $S_{5}$, we can see a huge difference in comparison to the other scenarios due to the reduction of the SN active time. This makes it harder for the SN and the UAV to connect with each other and forces the UAV to fly over longer distances between SNs in hibernation mode. It is important to highlight that there are scenarios with lower SN density that generate larger UAV path length, and some other scenarios with higher SN density that create smaller route lengths due to SN grouping.

**Figure 16:**
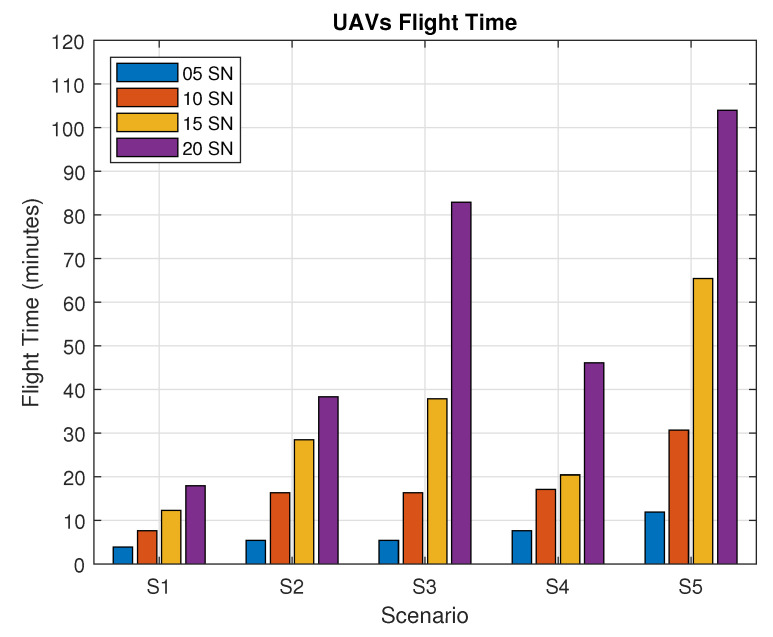
Flight time for each scenario.

Figure 16 shows the average UAV flight time for each scenario during the SN reconfiguration phase. The flight time in $S_{2}$ (with shared timeslots) did not differ significantly for 5 SNs in comparison with $S_{1}$; however, for 10, 15, and 20 SNs, we can see a significant difference on UAV flight time, due to its flying pattern over the other SNs even on timeslots that already been located other SNs. For $S_{3}$ (where the UAV waits over each SN), the average flight time of the UAVs is higher in comparison to $S_{1}$ for 10, 15, and 20 SNs as the UAV waits hovering over the SN as the timeslots pass by. For $S_{4}$, we recall that the UAV learning strategy is disabled; thus, it has higher average flight times when compared to $S_{1}$, for cases from 10 to 20 SNs. As for $S_{5}$, where the active SN transmission time is reduced to a single timeslot, the average flight time presents much higher values when compared to other scenarios; due to the difficulty of matching the SN active time, the UAV flies for longer.

**Figure 17:**
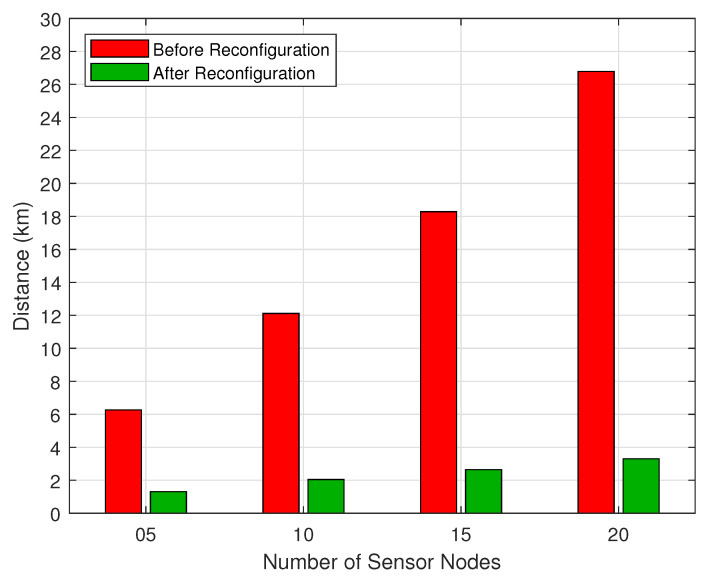
Flight distance during the reconfiguration phase.

The parameters presented in Table 1 for Scenario $S_{1}$ offered us the best performance based on our results compared to the other scenarios, so we performed some individual analysis to understand the behavior of our strategy. In Figure 17, we present the average distances covered by the UAVs during the reconfiguration phase along with the optimized route distance after the reconfiguration phase, for $S_{1}$. As we can see, for 10 SNs, the UAV needs to fly over an average of 12 km to locate them in proper timeslots; after this, the UAV can reduce its route to approximately 2 km. Still, according to the same figure, the difference is even more significant for denser scenarios: for 20 SNs, the UAVs traveled on average 27 km until reconfiguring all SNs. In contrast, after the reconfiguration phase, the route is reduced to just 4 km.

**Figure 18:**
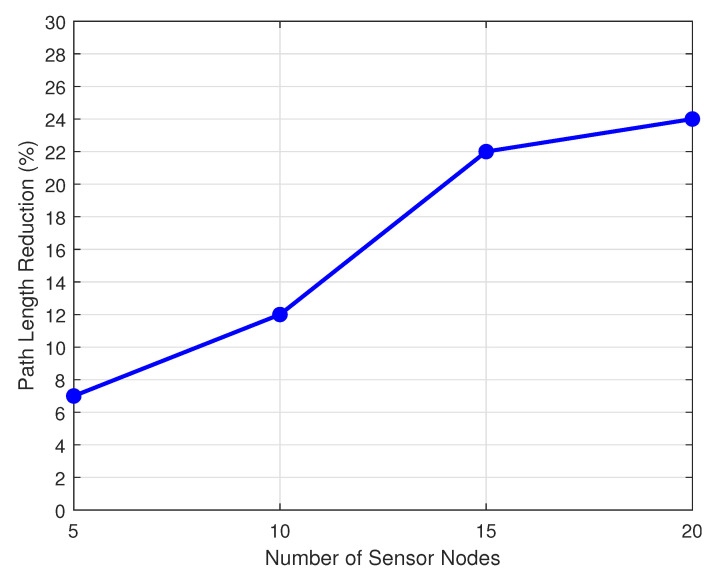
Reduction in traveled distance of $S_{1}$ compared to $S_{4}$.

Figure 18 compares the reduction in traveled distance obtained in scenario $S_{1}$ when compared to $S_{4}$, in order to measure the impact of the UAV decision-making strategy. The reduction in traveled distance is as great as 20% for scenarios with 15 and 20 SNs. In scenarios where the UAV can make a decision, it flies to another SN if it determines that the arrival TS is restricted to that destination. If it has no possible destination, the UAV simply waits on its current position until the next TS, avoiding unnecessary traveling.

**Figure 19:**
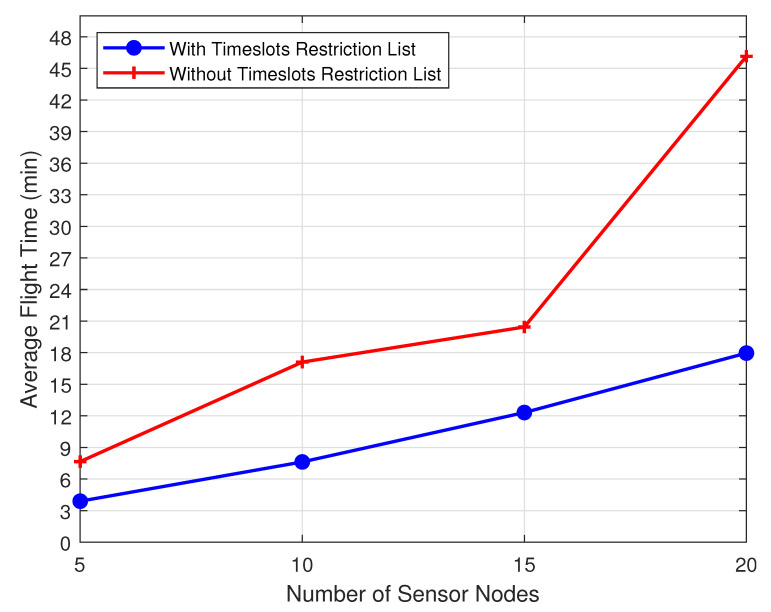
Flight time with and without Prohibition List.

Figure 19 presents a flight time comparison between scenarios $S_{1}$ and $S_{4}$, taking into account the use or not of the learning system based on the Prohibition List that the UAV feeds during the SN reconfiguration phase. Considering the case of 20 SNs, we can observe a reduction in the average flight time from 46 min to 17 min (savings of 63%) to complete the reconfiguration phase. Even for less dense cases, such as with 5 and 10 SNs, we obtain more than 53% of reduction in the UAV flight time.

**Figure 20:**
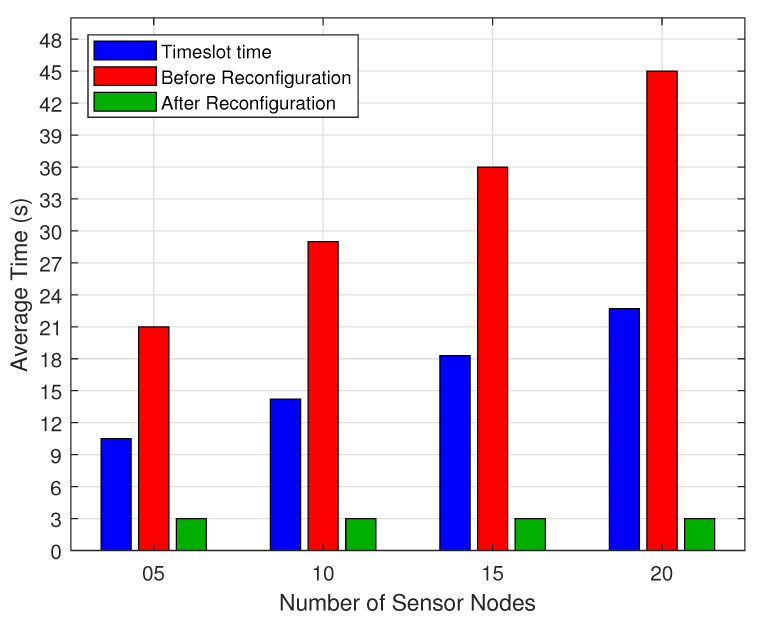
Active transmission time before and after reconfiguration.

Figure 20 shows a comparison of the SNs average active time before and after the reconfiguration process for the scenario $S_{1}$. After that, the UAV is able to position itself over the SN coordinates for a short time before the SN goes into active mode in order to collect sensory data. Once detecting that the SN is in active mode, the UAV collects the sensory data for a period—here called Reading Time ($t_{R}$)—and then forces the SN to hibernate again until its next active timeslot. In all simulations, we adopt the value of $t_{R}=3$ s, as shown in Table 1. For scenarios with 20 SNs and timeslot time ($N_{\tau }$) = 22 s, we obtained a reduction of up to 89% in the SN active time after scenario reconfiguration, which is a fundamental factor to guarantee a longer lifetime of the SNs.

**Figure 21:**
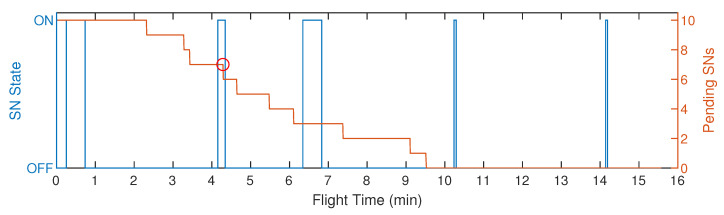
Graphical representation of an SN active and hibernation states, considering $S_{1}$ with 10 SNs.

Figure 21 presents an analysis of the hibernation and active states from a single SN, considering $S_{1}$ with 10 SNs. The representation of other SNs was omitted to facilitate the analysis. On the left vertical axis, we have the SN states (active is represented as ON and hibernation is OFF). On the right vertical axis, we have the amount of pending SNs, waiting to be reconfigured by the UAV. Both axes are in function of the UAV flight time. For scenarios $S_{1}$ to $S_{4}$, we have a particularity, where the SN active time is configured for two consecutive timeslots, facilitating the rendezvous of an SN by the UAV. On the other hand, it means that there is a higher energy consumption from the SN. According to the analysis and the graphical representation generated by the logs in Figure 21, we can see that the SN transmitted for 29 s (two consecutive timeslots) in the first minute of UAV flight; however, it was not found by the UAV. Then, at 4 min, we can see that the SN active period is shorter than the first one; furthermore, according to the graph represented by the right vertical axis, we can see a reduction in the number of pending SNs at this moment (red circle indicated in the graph), which characterizes that this SN was located and reconfigured by the UAV, that is, one less pending SN. We recall that, when the UAV reconfigures or collects the sensory data from any SN, it forces them to hibernate until its next activation TS, shortening its active time, which is why the activation period was shorter. Between 6 and 7 min, the SN active period were two timeslots, it is because the UAV removed this SN from its reconfiguration route. After 10 min of flight, we can notice that there are no more SNs to reconfigure, since the number of pending SNs is zero—that is, all SNs are already transmitting according to the optimized UAV route. Therefore, the UAV will always be positioned over the SN when its state becomes active, collecting its sensory data for the configured period of Reading Time ($t_{R}$) configured for 3 s, then putting the SN to hibernate until its next activation round. Therefore, we can graphically observe a significant reduction in the SN active period, after the UAV finished to locate all SNs (pending SNs equal to zero).

**Figure 22:**
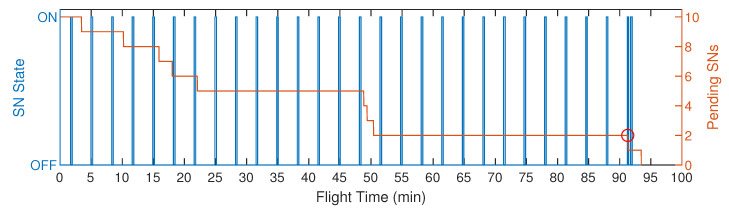
Graphical representation of an SN active and hibernation states, considering $S_{5}$ with 10 SNs.

Figure 22 presents a similar analysis but for $S_{5}$ with 10 SNs, the worst case scenario at the point of view of the SNs reconfiguration time, as they are active during a single timeslot (14s). In the figure, we can observe the activation periods of a single SN until it is located by the UAV. The periods of the other SNs are not shown, in order to facilitate the analysis. The highlighted circle in the graph represents the moment when the SN is finally located and reconfigured by the UAV. The SN went into an active state for 27 times until the UAV reconfigure it, while the process itself took approximately 95 min. Furthermore, we can see that the UAV spent long periods flying over the SN without locating it in active mode. Between 22 and 46 min of flight time, the UAV flew among only five SNs. In addition, between 51 and 90 min, the UAV flew between only two SNs without locating them. This problem occurred because the UAV flew between the SNs as they were changing their states as it got close to them. Thus, their active time was too quick for the proper displacement of the UAV. As long as the UAV made decisions to wait at its own place, or to go to another SN on its route; it was getting close to find out the SN in active mode. Therefore, the proposed decision strategy is fundamental to assist the UAV task of localizing all the SNs in the active state in a more expedited manner, saving time and energy.

## 6. Conclusions

In this work, we proposed a method for data collection from SNs by an UAV for use in precision agriculture applications. The introduced Prohibition List strategy used by the UAVs allowed us to save 63% in flight time with respect to the case where this strategy was not used, guiding the UAV efficiently among SNs during the reconfiguration phase. This fact makes the system highly adaptable to changes in wireless sensor networks, thus favoring the decision-making process by guaranteeing the freshness and continuity of the gathered data. Regarding the distance covered by the UAVs, we obtained a reduction of more than 20% in their flight path, due to their decision-making in terms of going or not to not yet visited SNs, which made the process more accurate. In relation to the SN, we obtained a significant reduction of 89% over its active time with the proposed strategy, minimizing it to only the reading time. This was done by positioning the UAV only a few moments before the SN becomes active and forces it to hibernate until its next cycle after the data collection.

Future work may consider, for instance, the impact of SN battery depletion, so that the UAV must detect that a device is no longer operational and therefore recalculates the optimized route. A dual problem is the detection of new devices and consequential route recalculation. Other interesting issue is to consider more than one UAV simultaneously flying over the SNs, which allows the design of a collaborative system, exchanging messages among UAVs for discovering SNs. Another extension is to consider the effect of optimizing transmission rate and power at each SN, which may considerably impact the battery lifetime and flight time.

## Figures and Tables

**Table 1:** Specification of Simulation Scenarios.

Global Parameters					
Path Planning Algorithm	DDNN				
Number of sensor nodes (SNs)	5, 10, 15 and 20				
SN Communication Range	100 m				
Simulation Area	1200 m × 500 m				
Number of Timeslots (TS)	TS ≥ 10				
UAV Flight Autonomy	600 s				
Reading Time ($t_{R}$)	3 s				
**Variable Parameters**	**$S_{1}$**	**$S_{2}$**	**$S_{3}$**	**$S_{4}$**	**$S_{5}$**
UAV Average Speed (m/s)	25	25	Wait	25	25
Shared or Dedicated Timeslots	Dedicated	Shared	Dedicated	Dedicated	Dedicated
SN Active Time (timeslots)	2	2	2	2	1
Prohibition List	Active	Active	Active	Inactive	Active

**Table 2:** Comparative methods from other projects.

	Method	Observations
Proposed work	Pre-programming of each SN timeslot and UAV sends rendezvous messages to ensure SNs timeslots sync.	We assumed that each SN is in a random timeslot, and the UAV is used as a sink node to collect data and to reconfigure it accordingly its own route; Our target is the dynamics of reconfiguration process (decisions, restriction list and flight length/time)
Trotta et al. (BEE-Drones) [20]	UAV uses a wake-up transmitter and installed a wake up receiver in SNs; Authors displaced wireless charging stations over the area to charge UAVs.	Results based on number of readings and quality of collected data; Clusters the SNs into different groups to each UAV; After that applies path planning.
Xiong et al. (DroneTank) [21]	Assume timeslots are assigned to each SN Uses an algorithm called Watertank to manage SNs active timeslots ensuring synchronization with UAV path. Watertank ensures the closest SN from UAV will be able to transmit its data, reducing energy budget.	Only natural landscapes (river, power transmission lines, ...); Do no explain how timeslots are assigned to SNs; Takes curves into consideration and UAVs energy budget for that; Results are based on data collection in function of network size, average energy from SN, wireless tx range; Focus on energy budget from UAV and SN, and data delivery
Zhan et al. [22]	Downlink to wake up SNs Considers only four SNs. Based on individual timeslots, and there is always one SN active per timeslot.	They consider that at least one SN is awake every timeslot to communicate with UAV; There is a downlink control to communicate with SN that wake them up while UAV flies over them; Considers few SNs, only 4. Conclusions based on wake-up time, consumption and rate

**Table 3:** Descriptive results obtained on simulations.

	UAV’s Battery Consumption				Decisions				Flight Distance (km)				Flight Time (min)			
	5	10	15	20	5	10	15	20	5	10	15	20	5	10	15	20
$S_{1}$	0.39	0.76	1.23	1.80	0.71	2.32	5.43	7.77	6.27	12.12	18.29	26.79	3.89	7.62	12.31	17.96
$S_{2}$	0.54	1.64	2.85	3.83	0.64	3.25	7.30	10.38	6.76	13.80	23.36	35.47	5.45	16.36	28.50	38.31
$S_{3}$	0.54	1.64	3.79	8.29	0.00	0.00	0.00	0.00	1.81	4.06	7.13	12.70	5.45	16.36	37.91	82.91
$S_{4}$	0.77	1.71	2.04	4.62	0.00	0.00	0.00	0.00	8.02	16.10	20.65	32.18	7.65	17.11	20.44	46.16
$S_{5}$	1.19	3.07	6.54	10.40	5.52	12.16	18.83	30.71	10.98	21.07	31.14	50.74	11.91	30.74	65.42	103.98
